# Controlled Human Malaria Infection in Semi-Immune Kenyan Adults (CHMI-SIKA): a study protocol to investigate *in vivo Plasmodium falciparum *malaria parasite growth in the context of pre-existing immunity

**DOI:** 10.12688/wellcomeopenres.14909.2

**Published:** 2019-11-14

**Authors:** Melissa C. Kapulu, Patricia Njuguna, Mainga M. Hamaluba, CHMI-SIKA Study Team

**Affiliations:** 1 KEMRI-Wellcome Research Programme, Kilifi, Kenya; 2 Centre for Tropical Medicine and Global Health, Nuffield Department of Medicine, University Oxford, Oxford, UK

**Keywords:** Plasmodium falciparum, controlled human malaria infection, PfSPZ Challenge, immunity, blood-stage, Kenya, parasite growth, quantitative PCR

## Abstract

Malaria remains a major public health burden despite approval for implementation of a partially effective pre-erythrocytic malaria vaccine. There is an urgent need to accelerate development of a more effective multi-stage vaccine. Adults in malaria endemic areas may have substantial immunity provided by responses to the blood stages of malaria parasites, but field trials conducted on several blood-stage vaccines have not shown high levels of efficacy.  We will use the controlled human malaria infection (CHMI) models with malaria-exposed volunteers to identify correlations between immune responses and parasite growth rates *in vivo*.  Immune responses more strongly associated with control of parasite growth should be prioritized to accelerate malaria vaccine development. We aim to recruit up to 200 healthy adult volunteers from areas of differing malaria transmission in Kenya, and after confirming their health status through clinical examination and routine haematology and biochemistry, we will comprehensively characterize immunity to malaria using >100 blood-stage antigens. We will administer 3,200 aseptic, purified, cryopreserved *Plasmodium falciparum* sporozoites (PfSPZ Challenge) by direct venous inoculation. Serial quantitative polymerase chain reaction to measure parasite growth rate *in vivo* will be undertaken. Clinical and laboratory monitoring will be undertaken to ensure volunteer safety. In addition, we will also explore the perceptions and experiences of volunteers and other stakeholders in participating in a malaria volunteer infection study. Serum, plasma, peripheral blood mononuclear cells and whole blood will be stored to allow a comprehensive assessment of adaptive and innate host immunity. We will use CHMI in semi-immune adult volunteers to relate parasite growth outcomes with antibody responses and other markers of host immunity.

**Registration:** ClinicalTrials.gov identifier NCT02739763.

## Abbreviations

ACT: artemisinin combination therapy; ADRB: Antibody dependent respiratory burst assay; CHMI: controlled human malaria infection; CHMI-SIKA: controlled human malaria infection in semi-immune Kenyan adults; DVI: Direct venous inoculation; GIA: Growth Inhibition Assay; OPA: Opsonic Phagocytosis Assay; PfSPZ Challenge: Aseptic, purified, cryopreserved *P. falciparum* sporozoites; qPCR: quantitative Polymerase Chain Reaction; SPZ: Sporozoites.

## Introduction

Malaria remains a disease of global health importance, despite the gains made against reducing morbidity and mortality. The latest estimates of the burden of malaria indicate that 3.3 billion people are exposed with 216 million cases and over 445,000 deaths being reported, with Africa accounting for ~91% of deaths due to malaria ^1^. There has been encouraging progress made in some areas of Africa, but progress has now stalled ^2^. Elimination does not appear realistic in many areas with higher transmission ^3^, and progress is threatened by insecticide and drug resistance ^4, 5^. An effective vaccine strategy is required to deliver sustainable and cost-effective control ^6^. Sub-unit vaccine development to date has focused on a limited pool of empirically selected candidate antigens, and field trials have not shown high levels of efficacy ^7^.

## Malaria vaccine development

The current lead malaria vaccine, RTS,S, a sub-unit vaccine, is based on a single pre-erythrocytic stage antigen the circumsporozoite protein (CSP). It delivers ~30% protection with waning efficacy over a few years ^8, 9^. Whole *Plasmodium falcipaurm* (Pf) sporozoite (SPZ) vaccines have shown >90% protection against controlled human malaria infection (CHMI) and ~50% protection in the field ^10, 11^. Higher vaccine efficacy would be optimal. One approach would be addition of partner antigens from the blood or transmission stages of the parasite. There are currently few subunit candidate blood-stage vaccines in clinical development: candidate vaccines in clinical efficacy trials are based on only five different antigens, and none have shown high level efficacy against their primary endpoint ^12– 14^.

A critical step in the development of a subunit blood-stage vaccine is identifying which parasite antigens to prioritise as targets for vaccine development.

### Naturally acquired immunity to malaria to inform vaccine selection

One approach to understanding human immunity to malaria to date has been to identify correlates of immunity using cross-sectional surveys of children living in malaria endemic regions, identifying subsequent malaria episodes, and then linking potential correlates of host immunity to outcome ^15– 18^. However, the limitations of this approach include: (a) variation of exposure in the endemic population means some individuals are unexposed to infectious bites, but assessed as if they were “protected” ^19^; (b) parasite exposures are genetically heterogeneous ^20^; (c) heterogeneity of exposure even within a single endemic setting leads to confounding effects (i.e. higher exposure is causally linked to the covariate of interest, to other effector mechanisms of immunity, and also to risk of malaria episodes) ^21, 22^; and (d) the antibody responses of greatest interest may well be those that are infrequently raised by natural exposure and are therefore not well represented in a community cohort ^23^.

 By measuring the parasite growth rates among semi-immune adult volunteers from malaria-endemic regions taking part in controlled human malaria infection (CHMI) studies, we have the opportunity for a complementary measure of human immunity where higher levels of antibody than those generated in children can be studied, and where exposure is controlled thus avoiding the confounding factors described above.

### CHMI studies in semi-immune individuals

CHMI studies in developed countries have until recently depended on exposure to mosquito bites, which place logistic demands in terms of the incubation of blood-stage cultures, feeding and infection of laboratory reared mosquitoes in sufficient numbers, which then cannot be stored. Work in malaria endemic countries can be facilitated by using cryopreserved sporozoites. Hoffman and colleagues at Sanaria have addressed various technical challenges in developing this technology, including the need to prepare large numbers of aseptic, purified and viable Pf sporozoites ((SPZ) (Sanaria(R) PfSPZ Challenge)) which can be cryopreserved and injected by syringe when required ^24– 31^.

A number of studies have been conducted to date to establish the safety and efficiency of this approach ^24, 25^ and following a scale-up in production, CHMI studies are now possible at a very much greater scale ( Table 1). The direct venous inoculation (DVI) of sporozoites (PfSPZ Challenge) is the most efficient route of administration and provides a reproducible inoculum ^28, 29^. Recent studies have shown that the infection rate produced by intramuscular injection of 75,000 sporozoites (PfSPZ of PfSPZ Challenge) is comparable with 3,200 PfSPZ sporozoites administered by DVI and 5 mosquito infected bites in malaria-naïve volunteers. Thus there are fewer PfSPZ sporozoites required using the DVI route of administration to achieve the same infection rates observed by the intramuscular route of administration ^28^. The DVI route has further been proven to be safe and reliable resulting in infection of all volunteers administered ^29^.

**Table 1.** List of PfSPZ Challenge CHMI Studies in African Endemic Populations *.

Location	Study Type	Number of Volunteers	Route of Administration	Age (years)	Gender	Malaria Outcome ^1^	Reference
Equatorial Guinea	Vaccine efficacy Vaccine efficacy	52 104	DVI DVI	18–35 18–45	Both Both	TBS ^2^ TBS ^2^	NCT02859350 NCT03590340
Gabon	Infectivity Vaccine efficacy	20 12	DVI DVI	18–30 18–40	Both Both	TBS ^2^ TBS ^2^	34 PACTR201503001038304
Gambia	Infectivity	19	DVI	18–35	Males	qPCR	35
Kenya	Infectivity ^3^	28	IM	18–45	Both	TBS ^2^	31
Mali	Vaccine efficacy ^4^ Vaccine efficacy	62 45	DVI DVI	18–45 18–50	Both Both	TBS ^2^ TBS ^2^	NCT02996695 NCT02627456
Tanzania	Infectivity ^3^ Vaccine efficacy Vaccine efficacy Vaccine efficacy	24 64 24 18 ^^^	ID DVI DVI DVI	20–35 18–35 18–45 18–45	Males Males Both Both	TBS ^5^ TBS ^2^ TBS ^2^ TBS ^2^	30 36 NCT02613520 NCT03420053

*Current status as of 30th October 2019. ^1^Primary measure for malaria treatment/diagnosis; ^2^Studies included qPCR data for secondary parasitaemia analysis; ^3^Studies included dose optimisation for PfSPZ Challenge administration; ^4^PfSPZ Challenge in the context of chemoprophylaxis using chloroquine (PfSPZ-CVac); ^5^Studies included qRT-PCR data for secondary parasitaemia analysis; and ^PfSPZ Challenge including HIV positive individuals (N=9). DVI, direct venous inoculation; IM, intramuscular; ID, intradermal; qPCR, quantitative PCR; and TBS, thick blood smear.

Overall, CHMI studies have been reported to be safe with only myocardial events having occurred in two naïve volunteers (from a total of over 2,000 volunteers who have undergone CHMI), one with prior cardiovascular risk factors and evidence of atherosclerotic infarction, and one without risk factors and a less clear clinical picture ^32, 33^. Furthermore, no participant in CHMI has thus far developed an illness meeting criteria for severe malaria. Thus overall, administration of PfSPZ in CHMI appears to be safe. To date, there are 12 CHMI studies that have been conducted or planned in Africa involving over 450 volunteers with varying degrees of malaria exposure across 6 countries ( Table 1).

## Ethical considerations for CHMI studies in endemic populations

CHMI studies raise ethical issues regarding volunteer safety and as such require that careful medical supervision and mitigation of the risks of challenge. CHMI studies in endemic populations are often carried out in in-patient settings requiring the volunteers to stay at study premises for a considerable amount of time to ensure safety and to prevent acquiring natural malaria infections ^34, 36^. There has been extensive consideration of the appropriate financial compensation ^36^. Although volunteers would be motivated to participate for altruistic reasons, care should be observed to ensure that the levels of payments offered are not an undue influence which would cause the volunteers’ judgement to be unduly influenced and therefore minimise the risks and discomforts involved ^36^. However, CHMI studies may require that volunteers are isolated from others, leading volunteers to incur substantial expenses and inconvenience, and these expenses must be compensated for to ensure that volunteers are not financially worse off as a result of participating. The informed consent process requires that the participant understands that they can withdraw from the study at any point with no penalty or interference ^36, 37^. Nesting ethical and social empirical work within the CHMI study, we have recently shown the importance of examining participant perceptions in volunteering in CHMI studies ^38^ and aim to further explore experiences with a larger sample set and other stakeholders. 

## Study protocol

This study protocol has been written in accordance with the SPIRIT guidelines for reporting clinical trials. A completed SPIRIT checklist is available at the Harvard Dataverse ^39^.

### Study aim and objectives

CHMI-SIKA study will investigate how the *in vivo* parasite growth rate of *Plasmodium falciparum* (Pf) is modified by pre-existing immunity measured by antibody levels to blood-stage antigens.

### Primary objective

The primary objective is to measure the correlations between antibody levels to defined and well characterised malaria antigens and growth rates of *P. falciparum* in volunteers undergoing CHMI.

### Secondary objectives

The secondary objectives of the CHMI-SIKA study are:

- To confirm the safety of CHMI administered by DVI in semi-immune volunteers.
- To measure parasite growth rates in semi-immune volunteers.
- To establish a sample set for the study of immunity to malaria and its effect on parasite growth following CHMI in semi-immune volunteers.
- To explore the understanding, motivations for participation, and experiences of volunteers and other stakeholders.

### Study design

**Figure 1.**
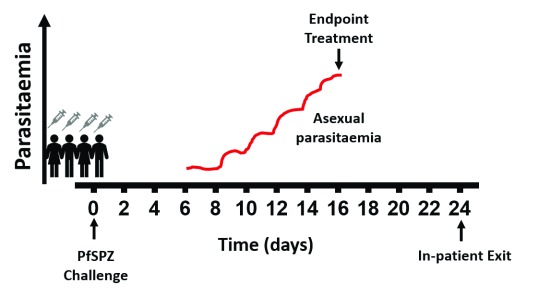
Schematic of CHMI PfSPZ challenge study profile.

CHMI-SIKA is an open label, non-randomised trial involving 200 healthy volunteers aged from 18 to 45 years recruited over a period of 5 years with varying degrees of immunity from malaria endemic areas in Kenya. CHMI will comprise the direct venous inoculation of aseptic, purified, cryopreserved PfSPZ (Sanaria(R) PfSPZ Challenge). Volunteers will be enrolled for PfSPZ Challenge in groups of 20–60 volunteers per CHMI event (henceforth referred to as CHMI cohorts) at any given time. We will screen all volunteers for varying degrees of immunity, ensuring a range of volunteers with low and high antibody responses to schizont extract. Volunteers will also be screened for significant medical conditions before enrolling in CHMI. Blood-stage malaria infection after 6.5 days of incubation in the liver will be assayed twice daily to monitor the density of infection and anti-malarial treatment will be given either: (a) when the density of infection rises past a threshold of 500 parasites per µl (a threshold substantially lower than 2,500 parasites per µl at which clinical illness becomes more common in children in Kenya ^40^) without signs and symptoms; (b) if a volunteer develops symptoms or signs of illness and an immediate blood film examination shows any evidence of detectable malaria parasites; or (c) the volunteer reaches day 21 of monitoring, at which point CHMI will be completed ( Figure 1). During the duration of CHMI, eligible volunteers will be accommodated at an in-patient setting where the volunteers will stay for the entire duration of the challenge ( Figure 1). All volunteers will be provided with and required to use an ITN for the duration of the study.

### Recruitment

The target volunteer population will be healthy, adult volunteers. Recruitment will target residents of Ahero in Kisumu County, Western Kenya and residents of Kilifi County, Coastal Kenya by a team of community health workers and fieldworkers respectively. Investigators will emphasize that participation in the study is voluntary. Participant information sheets will be given to interested volunteers. Individuals who feel that the trial is potentially appropriate for them will be invited to attend a formal screening visit with a study clinician. Recruitment in Kilifi will target residents within Kilifi North (Ngerenya) and Kilifi South (Pingilikani and Junju) sub-locations of Kilifi County, where malaria transmission is low to moderate ^41^. Recruitment in Kisumu will target residents of Ahero where transmission is high ^42^.

In Kilifi, community wide meetings to inform potential volunteers about the study will be conducted in the sub-locations followed by meetings with smaller groups of potential volunteers who are interested in participating by fieldworkers. In Ahero, community health workers will conduct meetings with smaller groups of potential volunteers. Volunteers from Ahero will have to be fluent in Kiswahili and/or English, as they will be asked to travel to Kilifi for the challenge event.

Recruitment for the social science component will involve only volunteers who are recruited, consented, and eligible to be enrolled for PfSPZ Challenge administration. Following recruitment to the CHMI study, volunteers will undergo a separate consenting process ^38^ to take part in the social science arm of the study. In addition, relevant stakeholders involved in the study including study clinical and research staff, community representatives and/or field workers will be recruited and consented for participation in this aspect of the study. All volunteers in this part of the study will be recruited after a process of information giving about this component of the study. All consent forms are available to view on Harvard Dataverse ^39^.

### Screening

All potential volunteers will have a screening visit either at the clinical facility in Ahero (Ahero Clinical Trials Unit, ACTU) or in Kilifi at the Kenya Medical Research Institute Wellcome Trust Research Programme (KEMRI-WTRP) which may take place up to 120 days prior to enrolment into a CHMI cohort. The screening visit will involve assessment of malaria past exposure using both laboratory tests and history. Informed consent will be taken before screening. If consent is obtained, the screening procedures indicated in the schedule of procedures ( Table 2) will be undertaken. Abnormal clinical findings from the medical history, clinical assessment or blood tests at any point in the study will be assessed. If a test is deemed clinically significant it may be repeated to confirm the result. If an abnormal finding is deemed to be clinically significant, the volunteer will be informed and referral to an appropriate medical centre arranged with the permission of the volunteer. Decisions to exclude the volunteer from enrolling in the trial or to withdraw a volunteer from the trial will be at the discretion of the Investigator.

**Table 2.** Schedule of Study Procedures.

Timeline (days in relation to challenge)	Screening	C-1	C	C+1 – C+24	C+35
Window (days)	-120 to -3	0	0	0	±5
ICF and ICF evaluation	X				
Medical History	X				
Clinical Assessment	X	X	X	X	X
Urinalysis	X				
Urinary β-hCG	X	X		X	
ECG	X				
Measurement of Height and Weight		X			
Administration of PfSPZ Challenge			X		
Local and Systemic events reviewed		X	X	X	X
Anti-malarial treatment directly observed				X	

C, PfSPZ Challenge; C-1, day before PfSPZ challenge; C+1, day 1 after PfSPZ challenge; β-hCG, β-human chorionic gonadotrophin; ECG, electrocardiogram. Clinical assessment will be based on tests conducted from blood samples collected.

### Selection of volunteers

***Inclusion criteria***

- Healthy adults aged 18 to 45 years.
- Able and willing (in the Investigator’s opinion) to comply with all study requirements.
- Informed consent.
- Use of effective method of contraception for the duration of the study (women only). We will ask female volunteers to come with their family planning records to verify. Effective contraception is defined as a contraceptive method with failure rate of less than 1% per year when used consistently and correctly, in accordance with the product label. Examples of these include: combined oral contraceptives; injectable progestogen; implants of etenogestrel or levonorgestrel; intrauterine device or intrauterine system; male partner sterilisation at least 6 months prior to the female subject’s entry into the study, and the relationship is monogamous; male condom combined with a vaginal spermicide (foam, gel, film, cream or suppository); and male condom combined with a female diaphragm, either with or without a vaginal spermicide (foam, gel, film, cream, or suppository).

***Exclusion criteria***

Any of the following constitutes exclusion criterion:

- Use of systemic antibiotics with known antimalarial activity within 30 days of administration of PfSPZ challenge (e.g. trimethoprim-sulfamethoxazole, doxycycline, tetracycline, clindamycin, erythromycin, fluoroquinolones and azithromycin).
- Receipt of an investigational product in the 30 days preceding enrolment, or planned receipt during the study period.
- Current participation in another clinical trial or recent participation within 12 weeks of enrolment.
- Prior receipt of an investigational malaria vaccine.
- Previous receipt of malaria sporozoites (PfSPZ) as part of the malaria challenge study.
- Any confirmed or suspected immunosuppressive or immunodeficient state, including HIV infection; asplenia; recurrent, severe infections and chronic (more than 14 days) immunosuppressant medication within the past 6 months (inhaled and topical steroids are allowed).
- Use of immunoglobulins or blood products within 3 months prior to enrolment.
- Any serious medical condition reported or identified during screening that increases the risk of CHMI.
- Any clinically significant abnormal finding on biochemistry or haematology blood tests, urinalysis or clinical examination.
- Women only; pregnancy, or an intention to become pregnant during the duration of the study.
- Confirmed parasite positive by PCR a day before challenge i.e. at C-1.

Exclusion criterion on day of PfSPZ Challenge administration:

- Acute disease, defined as moderate or severe illness with or without fever (temperature >37.5°C).

### Enrolment

We will enrol up to a total of 200 volunteers in CHMI. All volunteers will give written informed consent before being enrolled, after having been informed of the nature of the study, the potential risks and their obligations. We will conduct the CHMI studies in groups of 20 to 60 volunteers per cohort as guided by logistic and operational considerations.

***Consent***. All volunteers will sign and date the informed consent form before any study specific procedures are performed. The clinician or investigator will have a checklist for them to discuss with the potential volunteer aimed to assess their understanding of the study. The information sheet will be made available to the volunteer at least 24 hours prior to the screening visit. All informed consent documents will be translated into local languages (Dholuo, Kiswahili and Giriama). Particular points to be covered will be:

- Participation in the study is entirely voluntary.
- Declining to participate involves no penalty or loss of medical benefits.
- A volunteer may withdraw from the study at any time.
- A volunteer is free to ask questions at any time to allow him or her to understand the purpose of the study and the procedures involved.
- There is no direct benefit from participating. The benefits will be realized in the long-term for the community by contributing towards the development of a malaria vaccine.
- Volunteers will be compensated for travel, time and inconvenience of participating

***Withdrawal criteria***. In accordance with the principles of the current revision of the Declaration of Helsinki (updated 2008) and any other applicable regulations, a volunteer has the right to withdraw from the study at any time and for any reason and is not obliged to give his or her reasons for doing so. The Investigator may withdraw the volunteer at any time in the interests of the volunteer’s health and well-being. In addition, the volunteer may withdraw/be withdrawn for any of the following reasons:

- Administrative decision by the Investigator
- Ineligibility (either arising during the study or retrospectively, having been overlooked at screening)
- Significant protocol deviation
- Volunteer non-compliance with study requirements

***Managing withdrawals***. The reason for withdrawal will be recorded in the study case report form (CRF) ^39^. If withdrawal is due to an adverse event (AE), appropriate follow-up visits or medical care will be arranged, with the agreement of the volunteer, until the AE has resolved, stabilised or a non-study related causality has been assigned. If a volunteer withdraws/is withdrawn from the study after receiving PfSPZ Challenge but before reaching the criterion for malaria diagnosis, a complete, appropriate dose of the recommended anti-malarials will be provided and directly observed by the study clinical team. The importance of taking this medication will be stressed to the volunteer. If a volunteer withdraws from the study, blood samples collected before their withdrawal from the trial will be used and stored. Data from volunteers withdrawn from the study before fulfilling the criterion for malaria diagnosis will be excluded from the analysis of results relating to the study’s primary objective. Data from volunteers withdrawn from the study after fulfilling the criterion for malaria diagnosis will be included in analysis of results relating to the study’s primary objective.

### Study procedures

Procedures will be performed at the time points indicated in the schedule of procedures in Table 2. Additional procedures or laboratory tests may be performed at the discretion of the investigators if clinically necessary (e.g. urine microscopy in the event of positive urinalysis). Observations including pulse rate, blood pressure, respiratory rate and temperature will be measured at the time points indicated in the schedule of procedures. In addition, for blood tests, blood will be drawn at various time points ( Table 2 and Table 3) and the following laboratory assays performed: haematology (complete blood count (CBC); biochemistry (assessing sodium, potassium, urea, creatinine, albumin, ALT and bilirubin); diagnostic serology for HIV antibodies, Hepatitis B and Hepatitis C; immunological assays of prior exposure to malaria including antibody and cell-mediated immunity; diagnostic malaria tests being primarily qPCR for *P. falciparum* DNA and microscopy, and for detection of parasite sexual stages using a reverse transcriptase PCR assay; and DNA Genotyping PCR for red blood cell polymorphisms including but not limited to sickle cell trait, alpha-thalassemia, Dantu mutation of glycophorin A, and others that might affect susceptibility or resistance to malaria ^43^. Urinalysis will be done for the presence of clinically significant proteinuria, glucosuria or haematuria at screening and at various follow-up time points. Urine will also be tested for β-human chorionic gonadotropin (β-hCG) in female volunteers at screening, prior to administration of PfSPZ challenge and prior to start of anti-malarial medication. Electrocardiograms (ECGs) will be performed at screening and examined by a clinically qualified investigator for evidence of heart disease.

**Table 3.** Schedule of blood samples and volumes (in ml) for Screening to day before PfSPZ Challenge.

Variable	Screening	Repeat PCR check (where applicable)	C-1
PCR ^$^	4	4	4
FBC	1		1
Biochemistry *	2		2
Serology (HIV and Hepatitis B) ^^^	1		
Whole blood in RNA stabilizing buffer ^#^	2		2
Plasma/PBMCs ^+^	10		50
Volume (ml)	20	4	59
Cumulative Total (ml)	20	24	83

C-1= day before PfSPZ challenge; ^$^Includes screening for sickle cell trait, alpha-thalassaemia and other red blood cell polymorphisms and for gametocyte detection; *Biochemistry will include sodium, potassium, urea, creatinine, albumin, ALT and bilirubin; ^for assessing antibody responses; ^#^for transcriptome analysis; ^+^for immune responses.

For HIV diagnostic testing, volunteers will have information provided prior to HIV test counselling. HIV sero-status will be established using the standard rapid diagnostic kits in the lab as per the testing algorithm used by the Kenyan Ministry of Health. Those diagnosed as HIV antibody-positive will be referred to an appropriate health centre for further counselling and treatment. To maintain the confidentiality of those volunteers infected with HIV, we will make it clear that during screening one can be excluded due to a range of conditions (not just HIV), as well as abnormal laboratory results.

***Malaria infection prior to PfSPZ Challenge***. In order to prevent pre-existing malaria infection from interfering with the study, eligible volunteers who are found to be PCR-positive for malaria infection at screening will be treated with 7 days of artesunate (this drug was chosen to avoid other drugs with long half-lives that might interfere with subsequent CHMI), and the volunteer will then be screened again by PCR to confirm that they are negative in the week before being included in CHMI. In the case where a time lag of 10 days or more occurs between screening and challenge, all screened volunteers will be treated with the 7-day artesunate observed dose and a repeat PCR screen undertaken at least three days before CHMI (C-3). CHMI will be undertaken during the dry seasons to avoid high rates of malaria re-infection leading to volunteers being ineligible.

***Preparation and administration of PfSPZ Challenge***. For preparation of PfSPZ Challenge, immediately prior to use, PfSPZ Challenge in cryovials will be thawed individually by partial submersion of the vials for 30 seconds in a 37±1°C water bath. Designated, trained study staff will then prepare, dilute and dispense PfSPZ Challenge to clinical staff. Aliquots of the diluents phosphate buffered saline (PBS) and 25% human serum albumin (HSA) will be provided to the clinical sites by Sanaria Inc. PfSPZ Challenge will be administered using a needle and syringe by DVI.

During administration of PfSPZ Challenge, advanced life support drugs and resuscitation equipment will be immediately available for the management of anaphylaxis. Volunteers will be observed for 1 hour after injection at the study clinic before returning to the in-patient setting located ~2.5km from the KEMRI Wellcome Trust Research Programme (KEMRI-KWTRP) at the local university (i.e. Pwani University). Following PfSPZ Challenge, volunteers will be resident as in-patients until the treatment criteria are met or they withdraw/are withdrawn. The injection sites will be covered with a sterile dressing. The sterile dressing will be removed no earlier than 1 hour after inoculation. Any unsolicited adverse events or serious adverse events (SAE) will be recorded. (See Safety Monitoring below).

We will inject 3,200 parasites DVI. This will be done by a trained clinician with observation of the injection technique from personnel experienced in PfSPZ injection. The dose and route of administration of PfSPZ Challenge to be used in this trial have been chosen to maximise the likelihood of successful infection with malaria and are based on data from previous trials of PfSPZ Challenge in malaria-naïve individuals and in malaria endemic regions (see Introduction).

***Monitoring after PfSPZ Challenge***. A day before (C-1) administration of PfSPZ Challenge, all eligible volunteers will be asked to report to the study clinic to be re-assessed. All volunteers will be required to be enrolled at an in-patient setting near the study clinic in Kilifi until completion of endpoint anti-malarial treatment. Assessment of any new medical conditions or symptoms that have arisen since screening will be performed. Clinical assessments (including measurement of height and weight), urinary β-hCG and blood tests will be undertaken according to Table 3 and Table 4. Results of blood tests taken at this visit will be made available and reviewed prior to PfSPZ Challenge administration.

**Table 4.** Schedule of blood samples from challenge to challenge +35 days.

Days after challenge	5	7	7.5	8	8.5	9	9.5	10	10.5	11	11.5	12	12.5	13	13.5	14	14.5	15	16	17	18	19	20	21	Diag ^&^	+24hr	+48hr	+72hr	C+35
PCR		**4**	**4**	**4**	**4**	**4**	**4**	**4**	**4**	**4**	**4**	**4**	**4**	**4**	**4**	**4**	**4**	**4**	**4**	**4**	**4**	**4**	**4**	**4**	**4**	**4**	**4**	**4**	
FBC		**1**														**1**								**1**	**1**			**1**	**1**
Biochemistry * ^^^						**2**																		**2**	**2**				
Plasma/PBMCs ^$^	**30**	**30**				**30**										**30**								**30**	**30**				**50**
RNA analysis	**2**	**2**				**2**										**2**								**2**	**2**				
Parasite Typing																								**2**	**2**				
Vol (ml)	**32**	**37**	**4**	**4**	**4**	**38**	**4**	**4**	**4**	**4**	**4**	**4**	**4**	**4**	**4**	**37**	**4**	**4**	**4**	**4**	**4**	**4**	**4**	**41**	**41**	**4**	**4**	**5**	**51**
Cumulative ^#^ Total (ml)	**115**	**152**	**156**	**160**	**164**	**202**	**206**	**210**	**214**	**218**	**222**	**226**	**230**	**234**	**238**	**275**	**279**	**283**	**287**	**291**	**295**	**299**	**303**	**344**	**-**	**348**	**352**	**357**	**412**

Only one blood samples will be drawn between day of PfSPZ Challenge and C+6 (C+5). *Biochemistry will include Sodium, Potassium, Urea, Creatinine, Albumin, ALT & Bilirubin. ^Blood will be drawn for biochemistry on +9, C+21 and day of diagnosis. ^$^Blood will be taken for plasma and/or PBMCs on +5, +7, +9, +14, +21, and day of diagnosis and then not again until C+35. ^&^Blood sample will not be taken at diagnosis if a sample has already been taken on the same day. ^#^The cumulative total includes pre-challenge blood volumes indicated in Table 3 (i.e. 83ml + 32 ml to give first cumulative total of 115 ml).

On the day of PfSPZ Challenge administration, all volunteers will have clinical assessments performed prior to CHMI and review of all C-1 results. If withdrawal criteria are met this will be dealt with as described above. An hour after PfSPZ Challenge administration, volunteers will be monitored and any AEs occurring will be documented (solicited and unsolicited Table 5).

**Table 5.** Solicited adverse events related to malaria infection.

Adverse events	
Physical Signs	Fever
	Hypotension
	Tachycardia
Symptoms	Feverishness
	Chills
	Rigor
	Sweating
	Headache
	Anorexia
	Nausea
	Vomiting
	Myalgia
	Arthralgia
	Low Back Pain
	Fatigue
Laboratory Abnormalities	Lymphopenia
	Thrombocytopenia

The liver stage of malaria infection is asymptomatic and lasts 6 days. A blood sample will only be collected on day 5 post-PfSPZ Challenge (C+5), where a venous blood sample will be taken for assessment of liver stage immunity ( Table 4). During this period volunteers will be required to be resident at the in-patient setting and will have access to a study clinician and nurse in case of any symptoms. Between day one and four (C+1 – C+4), volunteers will be asked for their consent to participate in the social science component of the study. Those willing to participate will be asked to fill out a questionnaire regarding their expectations of the study and regarding the information they have received. In addition, 20–25 volunteers will be selected based on age, religion, and gender (to get a diversity of volunteers) and requested to participate in in-depth interviews and focus group discussions. All questionnaires are available on Harvard Dataverse ^39^.

During blood stage infection, between days 7 and 21 (C+7 to C+21), there will be daily review of the volunteers. At each review the following will be carried out:

- Clinical assessment.
- Volunteers will be questioned as to whether they have experienced any symptoms of malaria.
- Venous blood sampling will be performed as per schedule of attendance ( Table 4).
- Severity of symptoms will be assessed using grading criterion summarised in Table 6.

**Table 6.** Severity grading for adverse events.

Grading	Definition
Grade 0	None
Grade 1	Mild: Transient or mild limitation in activity (<48 hours); no medical intervention/therapy required
Grade 2	Moderate: Mild to moderate limitation in activity - some assistance may be needed; no or minimal medical intervention/therapy required
Grade 3	Severe: Marked limitation in activity, some assistance usually required; medical intervention/ therapy required, hospitalisation possible

Venous blood samples will be taken for qPCR for *P. falciparum* twice per day from days C+7 to C+14, and then once per day from C+15 to C+21. qPCR results will be processed within 6 hours of collection for the morning tests and within 18 hours for the evening tests. Endpoint anti-malarial treatment will be given based on qPCR results at a threshold of 500 parasites per µl (see study treatment below). A sensitive high-volume qPCR assay will be used for detection and any production of gametocytes using a reverse transcriptase PCR assay.

Following diagnosis, endpoint, volunteers will continue to be reviewed and have clinical observations performed once a day post-diagnosis. If qPCR results from blood taken at 24, 48 and 72 hours post-diagnosis are negative for parasites and the patient has no symptoms or mild, resolving symptoms, then the volunteer will be able to leave the in-patient setting and not reviewed again until C+35. If further blood samples are positive or symptoms are persistent then a medical assessment will be conducted, and further investigation or treatment planned according to the findings. On the last day of anti-malaria treatment, a blood sample will be drawn for complete blood count ( Table 4) for safety monitoring.

All volunteers will be required to attend a review clinic visit 35 days after challenge administration. Clinical assessments will be performed, and AEs assessed. Venous blood samples will be collected ( Table 4). In addition, a questionnaire will also be given during the C+35 visit to explore the perceptions of the volunteers regarding their experience in the study. For the in-depth interviews and discussions, 20–25 volunteers who were previously selected (during C+1 to C+6 above) will be requested to participate in more in-depth discussions.

### Study treatment

***Clinical reviews***. All clinical care and procedures will be undertaken by a qualified nurse and clinician trained in the study procedures. Following administration of PfSPZ challenge volunteers will remain at the in-patient setting until the endpoint treatment completion. Clinically qualified staff will be available at all times, and standard operating procedures (SOPs) will be established for out-of-hours assessment and management of symptoms. Volunteers and staff at the site will have contact numbers of clinically qualified investigators who in turn can consult a senior clinician at any time during CHMI.

The volunteers will be monitored by clinical staff with experience of managing clinical *P. falciparum* infection. Resuscitation equipment and anti-malarial drugs will be available at all times. The strain of parasite Pf, NF54 used for CHMI is known to be sensitive to chloroquine, artemether-lumefantrine (AL), atovaquone/proguanil and sulphadoxine-pyrimethamine (SP). These treatments are all known to be effective for uncomplicated malaria. A full treatment course will be given to all volunteers reaching the end of CHMI.

***Malaria management***. All volunteers will be treated with a full course of artemether-lumefantrine as endpoint anti-malaria treatment. Doses of treatment will be directly observed. The infecting parasites are known to be fully sensitive to artemether-lumefantrine. Endpoint anti-malarial treatment will be given based on qPCR results at a threshold of 500 parasites per µl. In addition, if a volunteer develops symptoms or signs of malaria then a rapid diagnostic test will be done and a sample taken for an immediate blood film examination to be conducted and results relayed immediately on availability. If any parasites are seen on the blood film then anti-malarials will be given. Blood films will be prepared by an experienced microscopist and 100 high power fields of both thick and thin films will be examined for parasites before the film is declared negative. The presence of a parasite will be confirmed by a second microscopist before the film is considered positive. Additionally, if the clinical investigators have concerns regarding the clinical condition of any volunteer they may advise treatment with anti-malarials irrespective of the results of PCR or microscopy. When a case of malaria is diagnosed, each subject will have a clinical evaluation by one of the investigators (a clinician) with appropriate history and clinical examination.

Volunteers who remain undiagnosed with malaria at C+21 will start a treatment course of artemether-lumefantrine at this time point. With this close observation we do not anticipate any serious illness developing. However, if in the opinion of a clinical investigator a volunteer shows signs that indicate the need for in-patient care then admission will be organized an appropriate hospital. If intensive care facilities are required or may be required, then a referral will be made to an appropriately equipped facility.

If a patient is unable to tolerate an oral anti-malarial, they will be treated with parenteral artesunate until they are able to take oral medication. If a volunteer withdraws/is withdrawn from the study after receiving PfSPZ Challenge but before reaching the criterion for malaria diagnosis, a complete, appropriate, curative course of anti-malarial therapy must be completed. The importance of this will be emphasised to volunteers at screening. If a volunteer develops a contraindication to artemether-lumefantrine or is unable to tolerate artemether-lumefantrine, oral chloroquine or SP may be prescribed as an alternative treatment for malaria. The strain of *P. falciparum* used is known to be sensitive to chloroquine, atovaquone/proguanil, SP, and artemether-lumefantrine. These drugs will be used in accordance with the manufacturer’s instructions and the Government of Kenya treatment guidelines.

### Safety monitoring

Safety oversight will be the responsibility of the investigators and the Data Safety Monitoring Committee (DSMC) that will be convened.

***DSMC***. A DSMC will be convened on behalf of the sponsor and will consist of 5 individuals who cover clinical and statistical expertise including at least 2 DSMC members based in Kenya. In addition, a member from Sanaria and one from Clinical Trials Facility in Kilifi will be available for the DSMC meetings to provide information and clarifications but will not be voting members on the DSMC and will not take part in the closed DSMC session. The DSMC will receive reports of all SAEs and suspected unexpected serious adverse reactions (SUSARs) as well as volunteers lost to follow up during CHMI. The DSMC will be empowered to stop some or all trial procedures by recommendation to the Sponsor. If such a recommendation is made, then research ethics committees will be informed within 3 working days of the recommendation. The DSMC Charter will be drawn before participant enrolment. Regular monitoring will be performed according to International Council for Harmonisation (ICH) good clinical practice (GCP) and a monitoring plan. Monitors will check whether the clinical trial is conducted, and data are generated, documented and reported in compliance with the protocol, GCP and the applicable regulatory requirements. The site team led by the PI will be responsible for local submissions to the research ethics committees and all the staff will have good clinical practice training prior to study start.

***Safety measures for PfSPZ Challenge***. Volunteer safety is of paramount importance. The following measures will be in place to safeguard volunteer safety:

- All volunteers will be asked to provide details of an emergency contact person who may be contacted if the volunteer cannot be contacted or located following CHMI and before treatment.
- All doses of artemether-lumefantrine will be observed by the study team. (For volunteers taking other anti-malarials, at least half of all doses will be observed).
- Volunteers will be counselled to contact the study team for review if they develop fever or other symptoms of malaria in the 6 months following the challenge.

***Adverse events (AEs)***. AEs will be documented in individual CRFs for each volunteer. They will be recorded under two headings; solicited and unsolicited. Any unforeseen and unavoidable deviations from the study protocol will be documented and filed in a protocol deviation folder, with explanation. Case report forms will be kept securely. The severity of AEs will be graded using the scale provided in Table 6. Events include:

1. Adverse events: Any untoward medical occurrence in a patient or clinical investigation subject occurring in any phase of the clinical study whether or not considered related to the vaccine. This includes an exacerbation of pre-existing conditions or events, intercurrent illnesses, or drug interactions. Anticipated day-to-day fluctuations of pre-existing conditions, including the disease under study, that do not represent a clinically significant exacerbation will not be considered AEs. Discrete episodes of chronic conditions occurring during a study period will be reported as adverse events to assess changes in frequency or severity.
  Unsolicited adverse events will be documented in terms of a medical diagnosis/diagnoses. When this is not possible, the AE will be documented in terms of signs and symptoms observed by the investigator or reported by the subject. Pre-existing conditions or signs and/or symptoms (including any which are not recognised at study entry but are recognised during the study period) present in a subject prior to the start of the study will be recorded on the medical history form within the subject's CRF.
2. SAEs: A SAE will be any untoward medical occurrence that at any time:
  • results in death,
  • is life-threatening,
  Note: The term “life-threatening” in the definition of “serious” refers to an event in which the patient was at risk of death at the time of the event; it does not refer to an event which hypothetically might have caused death if it were more severe.
  • Requires inpatient hospitalisation or prolongation of existing hospitalisation,
  • results in persistent or significant disability/incapacity, or
  • results in a congenital anomaly/birth defect.
  Medical and scientific judgment will be exercised in deciding whether expedited reporting is appropriate in other situations, such as important medical events that may not be immediately life-threatening or result in death or hospitalisation but may jeopardise the patient or may require intervention to prevent one of the other outcomes listed in the definition above. These will also usually be considered serious. SAEs related with drug treatment will not be reported to a sponsor, since the drugs are all licensed, but we will undertake expedited reporting to the DSMC and the ethics committees.
3. Suspected unexpected serious adverse reactions (SUSAR): An adverse reaction, the nature or severity of which is not anticipated based on the applicable product information is considered as an unexpected adverse drug reaction. Where the adverse reaction is also considered to have a possible, probable or definite relationship with the drugs given, and also meets the criteria for a serious adverse reaction, it is termed a SUSAR. These events are subject to expedited reporting as for SAEs.

***Reporting SAEs and unexpected AEs***. SAEs will be reported within 24 hours of their identification by study staff to the research ethics committees and DSMC. SUSARs will be reported within 1 working day to the same parties. If any volunteers are lost to follow up during CHMI without having completed a course of anti-malarials this will also be reported within 1 working day. The DSMC will be empowered to stop recruitment or study procedures if they believe it is required to protect the safety of volunteers. Adverse events likely to be related to CHMI, whether serious or not, which persist at the end of the trial will be followed up by the investigator until their resolution or stabilisation. The outcome will be assessed as: recovered/resolved; not recovered/not resolved; recovering/resolving; recovered with sequelae/resolved with sequelae; and fatal (SAEs only).

### Assessment of immunity to malaria

Immune responses, antibody and cell-mediated, will be assayed to determine signatures of immunity using plasma and peripheral blood mononuclear cells (PBMCs). For assessment of antibody immunological responses, techniques will include protein microarrays ^44^, antibody-dependent assays of functional immunity such as (GIAs) ^45– 47^, opsonic phagocytosis assays (OPA) ^48^, and antibody dependent respiratory burst (ADRB) ^46, 47, 49^. In further exploratory analysis we will also undertake principal component analyses to determine if there are typical “signatures” of protective responses, and analyses for combinations of protective antigens as previously described by Osier *et al*. 2014 ^17^. The statistical significance of protective antigen combinations will be tested using interaction terms and summary metrics as in previous studies ^17, 50^

A detailed analysis of cellular immune responses in PBMC, plasma and host transcriptomics will be undertaken and include the following: flow cytometry to determine cellular correlates of immunity; determination of the evolution of the T and B cell response at the transcriptomic and cellular levels; RNA sequencing and multiphoton microscopy to study the impact of exposure to parasites on dendritic cells; and determination of the role of antibody-mediated *P. falciparum* sporozoite inhibition. In addition, the parasite transcriptomes will be analysed as well as assessment of parasite isolates on the day of diagnosis to ensure that the signs and symptoms of malaria observed in the volunteers are not due to community-acquired infections but the infecting parasite strain, NF54.

Table 7 shows a list of proposed exploratory immunological assays to be performed on the samples generated from the study

**Table 7.** List of exploratory immunological assays to be performed on the samples generated from the study ^a^

Assay	Sample type	Reference
Antibody Dependent Respiratory Burst Assay (ADRB)	Plasma	43, 45, 47
Complement Fixation Assay	Plasma	51
DNA microarrays	Parasitised red blood cells (RBCs)	52, 53
Growth Inhibition Assay	Plasma	42, 43, 45
Mass Cytometry (CyTOF) ^1^	PBMCs	53, 54
Merozoite Opsonic Phagocytosis Assay (OPA)	Plasma	46
Multiphoton Microscopy ^2^	PBMCs	55, 56
Metabolomics	Plasma	57
Protein microarrays	Plasma	41
RNA Sequencing ^3^	PBMCs and parasitised RBCs	58
Sporozoite Inhibition Assay	Plasma	59

^a^Other assays will be conducted as and when they are readily available. ^1^Analysis of follicular helper T cells; ^2^Imaging studies of immune-modulatory effects on dendritic cell and T cell interactions; and ^3^T and B cell receptor repertoires including parasite transcriptomics.

***Sample use and storage***. This study will provide a platform for further studies to be conducted using the samples to be collected and stored. Additional ethics approval will be obtained for sub-analyses if different from primary and secondary objectives. The sample set is likely to be an important international resource for testing responses to new candidate antigens for vaccine development, and therefore we will need to store samples to allow further collaborations in the future. These collaborations will be supported if they are consistent with the protocol and as approved by the relevant research ethics committee. Testing outside the permission granted in this protocol will only be supported following an amendment or a new protocol. The samples collected will be stored in the KEMRI-Wellcome Trust Research Programme Kilifi Repository.

### Data management

The principal investigator (PI) will have overall responsibility for ensuring management of the data. A designee to the PI will be responsible for receiving, entering, cleaning, querying, analysing and storing all data that accrues from the study. Responsibility for this may be delegated to the study data management team. The data will be entered into the subjects’ paper screening CRF and study CRF ^39^. Data will be subsequently transferred to an electronic database for analysis. If any changes to the study are necessary during the study a formal amendment will be presented to the sponsor prior to submission to the relevant ethical and regulatory agencies for approval unless to eliminate an immediate hazard(s) to study participant without prior ethics approval. Any unforeseen and unavoidable deviations from the protocol will be documented and filed in as a protocol deviation in the Trial Master File, with explanation. A protocol deviation will be any failure to adhere to the defined procedures or treatment plans outlined in the protocol version previously approved by the relevant local research ethics committee. A protocol violation is any planned or inadvertent changes that may impact safety of study participants, affect integrity of the study data and/or affect study participants willingness to participate in the study previously approved by the relevant local research ethics committee. Both deviations and violations will be reported to the relevant local research ethics committee within 10 working days of the deviation/violation. The investigators will formulate a management plan to avoid the occurrence of a similar deviation in future.

***Data capture methods and archiving***. Data capture will be primarily via an offline database for the scheduled visits through use of computers and laptops. These data will then be transferred to an eCRF on Open Clinica. Paper source documents will be used to capture data for the screening visits, unscheduled clinic visits, laboratory and other investigational results. Data on scheduled visits may still be captured on paper source documents if electronic methods are not available. Immunological and PCR data will be transferred to an electronic database for analysis without any volunteer identifier apart from the unique volunteer number. For the social science component, the interviews and discussions will be recorded ^38, 39^. For archiving, the investigator will keep the consent forms and trial master file for at least 5 years after the completion or discontinuation of the trial. The anonymized electronic databases will be maintained beyond this period.

***Data sharing, reporting, and dissemination***. We will make the information on antigen prioritization publicly available and open access as rapidly as possible to provide the widest possible benefit of the study. We will feedback individual results with clinical relevance to volunteers in real-time. Summaries of the outcomes of the trial will be provided during community meetings in the areas from which volunteers are recruited. It is not anticipated that substantial information in this form will be available until at least the third year of recruiting, and this will be made clear during initial meetings to avoid unrealistic expectations regarding the rapidity of feedback.

### Statistics

***Sample size***. Sample size calculations were done considering power of the multivariable model using the powerreg facility from STATA 13, assuming an r ^2^ value of 0.3 for the combined model (i.e. including all variables) indicates 80% power to detect a single variable accounting for 0.15 of the variability in growth rates (after adjusting for other variables) on examining 200 volunteers when 50 variables are included in the model.

***Statistical and analytical plans***. Our analyses of these data will test the following hypotheses: (1) that naturally acquired immunity to malaria depends on responses to specific identifiable antigens; (2) that there are specific thresholds above which immunological responses control parasite growth; and (3) the overall intensity and/or breadth of antibody responses to parasite antigens are associated with immunity. These will be tested using generalized linear models using parasite growth patterns to derive the outcome variable. The parasite growth outcomes will be categorized by analysis of the PCR data and published prior to development of the statistical analysis for immune correlates.

There will be a deliberate analytical framework generated and applied to the data generated. However, this will be largely guided by the outcomes that will be observed and the results for e.g. qPCR. For instance, for parasite growth rates and liver-to-blood inoculums (i.e. the number of parasites exiting the liver and infected red blood cells), these will be calculated by fitting established models to quantitative PCR data. The growth rate will be regressed against the individual immunological measures such as intensity and/or breadth of antibody responses to parasite antigens. Given the co-linearity of responses, a multivariable model is likely to be more informative in testing our hypotheses that: naturally acquired immunity to malaria depends on responses to specific identifiable antigens; and the overall intensity and/or breadth of antibody responses to parasite antigens are associated with immunity. Rather than simply enter all variables in a single multivariable model, we will consider entering variables in clusters and take forward the independent predictors to a final multivariable model. These clusters will be pre-defined and be based on: (a) biological links (for instance grouping all red cell surface antigens separately from merozoite antigens); and (b) on co-linearity (i.e. on clustering groups of antigens with greatest cross-correlations). We will score breadth as the number of antigen responses in the top quartile and mean intensity as the mean of normalized responses for each antigen. These scores will be determined separately for merozoite antigens and for red-cell-surface antigens. We will therefore reduce the dataset to for instance 50 variables, including functional assays, anti-circumsporozoite antibody levels, responses to schizont extract, and scores for mean intensity and breadth which will be added to the final model which will test the hypotheses mentioned above. To test the hypothesis that there are specific thresholds above which immunological responses control parasite growth, we will examine this final model for non-linearity using multiple fractional polynomials and Hill functions to determine if there are discrete thresholds predicting efficacy according to previously used methods.

For the qualitative analysis, the recorded interviews will be transcribed and translated where necessary. The data will be organized and managed using NVIVO 10, a qualitative data management and analysis software. The data will be analysed using a thematic content analysis, which will allow for categorization of the recurrent and common themes and will allow for the presentation of the key elements of the volunteers’ accounts.

### Ethical compliance

***Study registration***. The study has been registered with the ClinicalTrials.gov registry ( NCT02739763).

***Ethical compliance***. Ethical approval has been granted by the institutional research ethics committee, Kenya Medical Research Institute Scientific and Ethics Review Unit (KEMRI/SERU/CGMR-C/029/3190) and by the sponsor of the study (University of Oxford) through the Oxford Tropical Research Ethics Committee (OxTREC, 2–16). The regulatory authority granted exemption of review for the study under the 2011 guidelines (Pharmacy and Poisons Board, PPB/ECCT/Misc/2015(61)).

***Confidentiality and data protection***. The clinical records will be kept in locked cabinets in the clinical trials facility. All immunological and qPCR data will be kept in anonymized databases linked by the study number to clinical data. For the qualitative social science component, all the recordings will be destroyed after the study has been completed. The data will be stored in password-protected computers and the hard copy documents will be stored in lockable cabinets. The interviews and discussions will be conducted in spaces that ensure privacy and confidentiality of the information provided by the volunteers as well as provide an environment that volunteers can freely express their opinions

***Community engagement***. Each site, Ahero CTU in Ahero and KWTRP in Kilifi will use existing community engagement strategies to inform communities about the study. For Kilifi, this will be the first CHMI study to be performed in the area and therefore there will be concerted effort together with the long-established community liaison group (CLG) to ensure that information giving, and community engagement activities are extensively carried out. A community engagement plan specific for the study will be developed between the CLG team and the investigators. Community meetings in areas of participant recruitment, meetings with chiefs, sub-chiefs, community representatives, and the Department of Health, Kilifi County will be organized to inform them about the study. Community engagement will continue throughout the study period of five years, collecting and responding to concerns from the community about the study through the CLG team.

***Publication policy***. Results will be published in an open-access journal. Anonymized data on PCR values and immunological data will be made available with these publications. The scientific and intellectual contributions of all persons involved in the research will be appropriately acknowledged in all publications and presentations arising from the work.

***Study status***. Ethical approval for CHMI-SIKA was granted in March 2016 and the study started enrolment in August 2016. To date (30th October 2019) we have enrolled 161 volunteers with two DSMC meetings. The foregoing protocol is written in the future tense reflecting that work is ongoing.

## Discussion

Malaria transmission is an ongoing public health problem in Africa. There is an urgent need to accelerate the development of a more effective vaccine. Accelerating the development of a malaria vaccine will be of substantial benefit. The controlled human malaria infection model in semi-immune adults helps overcome a critical block in vaccine development, i.e. comprehensive prioritization of antigens associated with blood-stage immunity for vaccine development. This will be the largest CHMI study in malaria-exposed individuals and will provide data relating to how naturally acquired immunity to malaria depends on specific responses and that overall intensity and/or breadth of antibody responses are associated with immunity. The outcome will determine antigen selection for the next generation of blood-stage malaria vaccines. In our experience of conducting these studies, we found it beneficial to embed social science framework studies to understand perceptions of such type of studies in endemic populations. We implemented this in the second cohort of recruitment and was considered in the first CHMI study conducted in Kenya ^35^. We drew on recommendations from Njue *et al*. ^38^ to shape and change some of the practices of conducting the study. Thus, this study will not only contribute new knowledge on potential correlates of protection in the context of naturally immunity, but the sample set generated will also allow for additional sub-studies to be performed to further unravel our understanding of immunity to malaria. Furthermore, this will also contribute to our understanding on how to undertake these types of studies through embedded social science sub-studies. This study also aims to build a stronger and more grounded understanding of the ethics of research using the CHMI model to inform future research in this context, and potentially in other similar settings.

## Data availability

The extended data have been uploaded to Harvard Dataverse under the title: “Replication Data for: Controlled Human Malaria Infection in Semi-Immune Kenyan Adults (CHMI-SIKA): a study protocol to investigate *in vivo Plasmodium falciparum* malaria parasite growth in the context of pre-existing immunity”. DOI: https://doi.org/10.7910/DVN/XOXLJQ ^39^.

The files available are listed below:

- Participant Information sheet and consent for social science sub-study – form 1 study participants
- Participant Information sheet and consent for social science sub-study – form 2 clinical/trial staff and field workers
- Participant Information sheet and consent for social science sub-study – form 3 community representatives
- Social Science sub-study entry questionnaire
- Social Science sub-study post-challenge questionnaire
- Case Reporting Form – screening
- Case Report Form - study
- Data Safety and Monitoring Committee Charter

Data are available under the terms of the Creative Commons Attribution 4.0 International license (CC-BY 4.0).

## Reporting guidelines

SPIRIT Checklist. DOI: https://doi.org/10.7910/DVN/XOXLJQ ^39^.

## Members of the CHMI-SIKA Study Team

Melissa C Kapulu ^1, 4^, Patricia Njuguna ^1^, Mainga Hamaluba ^1^, Abdirahman I Abdi ^1^, Yonas Abebe ^2^, Agnes Audi ^3^, Philip Bejon ^1, 4^, Peter Billingsley ^2^, Peter C Bull, Primus Che Chi ^1^, Zaydah de Laurent ^1^, Stephen Hoffman ^2^, Eric James ^2^, Irene Jao ^1^, Dorcas Kamuya ^1^, Gathoni Kamuyu ^1^, Silvia N Kariuki ^1^, Nelson Kibinge ^1^, Domtila Kimani ^1^, Rinter Kimathi ^1^, Sam Kinyanjui ^1, 4, 5^, Cheryl Kivisi ^5^, Nelly Koskei ^3^, Brett Lowe ^1, 4^, Johnstone Makale ^1^, Kevin Marsh ^1, 4^, Vicki Marsh ^1, 4^, Khadija Said Mohammed ^1^, Moses Mosobo ^1^, Linda Murungi ^1^, Janet Musembi ^1^, Jennifer Musyoki ^1^, Michelle Muthui ^1^, Jedida Mwacharo ^1^, Kennedy Mwai ^1^, Daniel Mwanga ^1^, Joyce Mwongeli ^1^, Francis Ndungu ^1^, Omar Ngoto ^1^, Maureen Njue ^1^, Irene Nkumama ^1, 6^, George Nyangweso ^1^, Dennis Odera ^1, 6^, Bernhards Ogutu ^3, 7^, Fredrick Olewe ^3^, James Oloo ^3^, Donwilliams Omuoyo ^1^, John Ongecha ^3^, Michael Ooko ^1^, Faith Osier ^1, 6^, Jimmy Shangala ^1^, Betty Kim Lee Sim ^2^, James Tuju ^1^, Juliana Wambua ^1^, Thomas N Williams ^1, 8^

## Affiliations

^1^KEMRI-Wellcome Trust Research Programme, Kilifi, Kenya

^2^Sanaria Inc., Rockville, MD, USA

^3^Centre for Clinical Research, Kenya Medical Research Institute, Kisumu, Kenya

^4^Centre for Tropical Medicine and Global Health, Nuffield Department of Medicine, University Oxford, Oxford, UK

^5^Pwani University, P. O. Box 195-80108, Kilifi, Kenya.

^6^Centre for Infectious Diseases, Heidelberg University Hospital, Heidelberg, Germany

^7^Center for Research in Therapeutic Sciences, Strathmore University, Nairobi, Kenya

^8^Department of Medicine, Imperial College, London, UK
