# Supplementary material for: Controlled Human Malaria Infection in Semi-Immune Kenyan Adults (CHMI-SIKA): a study protocol to investigate in vivo Plasmodium falciparum malaria parasite growth in the context of pre-existing immunity
Source: Wellcome Open Res. 2019 Nov 14;3:155. Originally published 2018 Dec 6. [Version 2] doi: 10.12688/wellcomeopenres.14909.2 (PMC6871356; doi:10.12688/wellcomeopenres.14909.2)
Supplement: Peer review history

# Peer review: Reviewer response for version 2

**DOI:** 10.21956/wellcomeopenres.17028.r37058

**Published:** 2019-11-18

**Authors:** Alister G. Craig

**Affiliations:** 1 Department of Tropical Disease Biology, Liverpool School of Tropical Medicine, Liverpool, UK

The authors have responded very well to my comments and have included all the information requested. This will be a complex study but the team appear to have the right structures in place to handle this. Pragmatically, there are limitations on defining "semi-immunity", particularly with regard to antigen recognition verses protection, and the study team are well-placed to handle these.

 I have no further comments.

I confirm that I have read this submission and believe that I have an appropriate level of expertise to confirm that it is of an acceptable scientific standard.

# Peer review: Reviewer response for version 1

**DOI:** 10.21956/wellcomeopenres.16254.r34926

**Published:** 2019-03-11

**Authors:** Salim Abdulla

**Affiliations:** 1 Ifakara Health Institute, Dar es Salaam, Tanzania

The investigators present a well-designed study using CHMI model to assess the impact of pre-existing antibodies on the blood stage replication of malaria parasites and hence use this indicator to prioritize which antigens to focus on in the malaria vaccine development effort. The investigators point out that so far the blood stage antigens in clinical development have shown modest efficacy and similarly with the sub-unit pre-erythrocytic vaccines. Better results have been seen with the use of whole sporozoite vaccine, generally thought to be due to the broader antigen profile presented as an immunogen. Hence, the idea of identifying other potent sub-unit antigens that can later be used in combination. The investigators mention that they will focus on the already known and well characterized antigens for this work.

 Comments and suggestions:

1. The use of the standard CHMI procedures as well as the social science component will further develop this approach for testing new candidate malaria diagnostics, drugs, vaccines and extend the application of the CHMI model into the exploration of the correlates of malaria immunity that is still not very well known.
2. It will be good for the investigators to provide a list of the specific antigens of interest in this study and describe a bit better how this approach of using CHMI platform to select for those antibodies that have been associated with reduced replication will be identified, different from previous work in which antibodies were similarly screened and episodes of parasitaemia monitored. Is the value of the CHMI only about removing the heterogeneity of malaria exposure in the assessment?
3. It will be good for the investigators to comment on the choice of using CHMI with Sporozoite Challenge inoculation approach while the interest is in blood stage antigens and a blood stage Challenge model exist that would have given a more uniform blood stage parasitaemia. I assume that one of the reasons apart from ease of procedures, is the breath of assessments that can be done using the Sporozoite Challenge approach. Example, pre-patent periods can be assessed as an indicator of liver stage activity. The investigators will take blood samples also during the liver stage replication of the parasites and hence using a battery of immunological assays already proposed to be done, be able to assess both pre-exiting as well as newly expressed antibodies for both liver stage as well as blood stage during the follow up period. How this information will be used to identify priority antigens is not very clear in the manuscript. Furthermore, observed high antibody titres does not always translate into effect in reducing replication so how are the other factors going to be teased out.
4. Is any work going to be done to identify novel antigens during this process?

I confirm that I have read this submission and believe that I have an appropriate level of expertise to confirm that it is of an acceptable scientific standard.

# Response

**Published:** 2019-10-30

**Authors:** Melissa Kapulu

**Affiliations:** KEMRI-Wellcome Trust Research Programme, Kenya

1. The use of the standard CHMI procedures as well as the social science component will further develop this approach for testing new candidate malaria diagnostics, drugs, vaccines and extend the application of the CHMI model into the exploration of the correlates of malaria immunity that is still not very well known.

 We thank the reviewer for their comment.

1. It will be good for the investigators to provide a list of the specific antigens of interest in this study and describe a bit better how this approach of using CHMI platform to select for those antibodies that have been associated with reduced replication will be identified, different from previous work in which antibodies were similarly screened and episodes of parasitaemia monitored. Is the value of the CHMI only about removing the heterogeneity of malaria exposure in the assessment?

 We have added the reference to the recently published KILCHIP paper Kamuyu et al 2018 describing the antigens. We anticipate that using the CHMI model in the context of pre-existing immunity will help identify potential antigens that could be prioritised for vaccine development. Heterogeneity of exposure is one of the factors that confounds analyses of association between immune responses and outcomes in the field. The CHMI model provides a “clean” system where individuals are equally exposed to a known quantity and genotype of parasites. The known timing of exposure also allows us to calculate the liver to blood inoculum in relation to parasite growth which would not be possible in cohort studies in the field.

1. It will be good for the investigators to comment on the choice of using CHMI with Sporozoite Challenge inoculation approach while the interest is in blood stage antigens and a blood stage Challenge model exist that would have given a more uniform blood stage parasitaemia. I assume that one of the reasons apart from ease of procedures, is the breath of assessments that can be done using the Sporozoite Challenge approach. Example, pre-patent periods can be assessed as an indicator of liver stage activity. The investigators will take blood samples also during the liver stage replication of the parasites and hence using a battery of immunological assays already proposed to be done, be able to assess both pre-exiting as well as newly expressed antibodies for both liver stage as well as blood stage during the follow up period. How this information will be used to identify priority antigens is not very clear in the manuscript. Furthermore, observed high antibody titres does not always translate into effect in reducing replication so how are the other factors going to be teased out.

We refer to our answer to reviewer number 1 comment 3 regarding the use of sporozoites. We agree with the reviewer that high antibody titres do not necessarily translate to a reduction in symptoms or effects on parasite growth. We will examine a wide range of antibody responses, in order to identify the limitations of seeking to explain outcomes with a single response.

1. Is any work going to be done to identify novel antigens during this process?

The antigens described on the microarray (Kamuyu et al 2018) include both previously characterized and uncharacterised and novel antigens. The use of this chip with these antigens is to examine their potential as vaccine candidates at the blood stage with complementary work on functional assays. There are plans to further use the sample set generated to identify “novel” antigens involved in the pre-erythrocytic and transmission stages of the parasite.

# Peer review: Reviewer response for version 1

**DOI:** 10.21956/wellcomeopenres.16254.r34397

**Published:** 2018-12-13

**Authors:** Alister G. Craig

**Affiliations:** 1 Department of Tropical Disease Biology, Liverpool School of Tropical Medicine, Liverpool, UK

This study protocol describes an approach to use a human malaria challenge model to identify lead candidates for blood-stage vaccines, based on screening semi-immune individuals for their ability to reduce parasite growth rates.  Many attempts have been made to identify this information from longitudinal studies of patient cohorts but, as the authors point out, these studies have a number of issues, particularly the specificity of the readouts against a wide variation in exposure to infection.  The authors seek to address this by using a group of volunteers who will be characterized for their immune status against a large panel of malaria antigens, and subsequently entered into a controlled sporozoite inoculation model of malaria infection to measure parasite growth rates during the erythrocytic cycles allowed in this model.

 Controlled human malaria infection (CHMI) is very well-established and provides an excellent platform to conduct experimental medicine research in a safe and acceptable format.  The authors follow a careful protocol for the CHMI phase and have access to experts in the field to guide them.  In terms of the CHMI aspect of this study protocol, I only have two comments:

1. From the protocol it seems that all volunteers will be treated in Kilifi, but recruited both from Kilifi and Kisumu, the latter being some 870km away.  This issue, in terms of how volunteers will be managed, is not discussed in the protocol.
2. The authors say that should intensive care facilities be needed, which is highly unlikely, “a referral will be made to an appropriately equipped facility”.  How far away from the testing centre is this facility?

My other comments relate to the study design with respect to the immune correlates:

1. An asexual parasite stage inoculation based CHMI model is also available.  Why are the authors using sporozoites, where there could be variability in the merozoite outputs from the liver stages, particularly in semi-immune individuals?  In some ways this can be controlled for by having the first cycle parasitaemia as a baseline so that growth rates can still be measured, but it may introduce variation in the erythrocytic ‘inoculum’ size.
2. The definition of ‘semi-immunity’ is not well described and it would be helpful to have some preliminary data on the levels of antibodies seen in the trial populations.  This would provide some structure to the decision on who will be recruited based on antibody levels.  At the moment the only comment on this is that a “range of volunteers with low and high antibodies to each antigen tested” will be recruited.
3. Along similar lines, from other vaccine trials and harmonized growth inhibitory assays (GIA), we now know that antigens vary in the level of antibodies needed to control parasite growth.  For example, the EC50s of MSP1, AMA1 and RH5 are 500ug/ml, 100ug/ml and 10ug/ml respectively.  Given this wide variation, how will the parameters for antibody/ antigen selection be derived?  Would there be a minimum cut-off below which a volunteer would be excluded from the trial as they would not be expected to show any control of parasite growth?  This is a complicated question as we think that antigens may not act independently in controlling parasitaemia, so lower level combinatorial effects cannot be excluded, but it may be prudent to remove very low responders to limit the amount of uninformative data and the risk to a volunteer from whom the expectation might be that the information derived from CHMI would not be useful in designing future vaccines (inasmuch as this can be predicted).
4. An appendix describing the antigens to be used for screening volunteers would be useful.  Will different variants of proteins be incorporated or just those for NF54 (the challenge strain)?
5. The range of analytical tools to be used to measure various aspects of host immunity is impressive, including phenotypic and transcriptional approaches.  A table to summarize these assays and provide references to their implementation elsewhere would be helpful in determined their likely efficacy.  Many of the assays are relatively common, but others are less so, at least to me (e.g. impact on dendritic cells; sporozoite inhibition).
6. The analytical part will generate a huge body of data, which provides two challenges:
  1. How will the database cope with the different forms of information?
  2. Does the power calculation take into account the multiplicity of parameters? 

Several of the authors have been involved in complex cohort studies on malaria immunity in Kilifi – have these generated an analytical framework that could be applied to this study?

1. Why are parasite transcriptomes being performed?  How will these data contribute to identifying immune signatures of protection?  If this is just to differentiate community acquired infection from the CHMI strain NF54, this can be done in a much less complicated way.

Finally, it is good to see the social science research nested within the project and these outcomes will provide useful information.  In terms of reporting this and other information, the authors say that they will make the data publicly available “as rapidly as possible”.  Would they be willing to put a time limit on this?

I confirm that I have read this submission and believe that I have an appropriate level of expertise to confirm that it is of an acceptable scientific standard, however I have significant reservations, as outlined above.

# Response

**Published:** 2019-10-30

**Authors:** Melissa Kapulu

**Affiliations:** KEMRI-Wellcome Trust Research Programme, Kenya

1. From the protocol it seems that all volunteers will be treated in Kilifi, but recruited both from Kilifi and Kisumu, the latter being some 870km away.  This issue, in terms of how volunteers will be managed, is not discussed in the protocol. 

Transport to Kilifi and back from Kilifi is organized and paid for by the research project. Participants from Kisumu will be screened for eligibility in Kisumu, then travel to Kilifi for participation in challenge, stay for the duration of the study, then return to Kisumu.

 

1. The authors say that should intensive care facilities be needed, which is highly unlikely, “a referral will be made to an appropriately equipped facility”.  How far away from the testing centre is this facility?

The facilities are in Mombasa are 65km away. We agree intensive care is highly unlikely to be required, although a very low threshold would be used for organizing early transfer to a hospital with intensive care facilities.

1. An asexual parasite stage inoculation based CHMI model is also available.  Why are the authors using sporozoites, where there could be variability in the merozoite outputs from the liver stages, particularly in semi-immune individuals?  In some ways this can be controlled for by having the first cycle parasitaemia as a baseline so that growth rates can still be measured, but it may introduce variation in the erythrocytic ‘inoculum’ size.

Cryopreserved sporozoites are GMP products that are standardized, produced at scale and acceptable to our community. There is a scientific advantage in being able to assess pre-erythrocytic immunity as well as blood-stage immunity. We agree with the reviewer that blood-stage challenge may reduce the variability in inoculum likely to be observed with sporozoites. We have not yet considered the acceptability of a blood product to our local community and would need to conduct further engagement to establish this.

1. The definition of ‘semi-immunity’ is not well described and it would be helpful to have some preliminary data on the levels of antibodies seen in the trial populations.  This would provide some structure to the decision on who will be recruited based on antibody levels.  At the moment the only comment on this is that a “range of volunteers with low and high antibodies to each antigen tested” will be recruited.

We do not believe there is an adequate definition of semi-immunity based on laboratory assays. As a pragmatic option we will use geographical location of residence and responses to schizont extract to stratify according to tertiles. The statement in reference to this under study design has been modified and clarified to read testing of antibodies to schizont extract, as follows: “We will screen all volunteers for varying degrees of immunity, ensuring a range of volunteers with low and high antibody responses to schizont extract.”

1. Along similar lines, from other vaccine trials and harmonized growth inhibitory assays (GIA), we now know that antigens vary in the level of antibodies needed to control parasite growth.  For example, the EC50s of MSP1, AMA1 and RH5 are 500ug/ml, 100ug/ml and 10ug/ml respectively.  Given this wide variation, how will the parameters for antibody/ antigen selection be derived?  Would there be a minimum cut-off below which a volunteer would be excluded from the trial as they would not be expected to show any control of parasite growth?  This is a complicated question as we think that antigens may not act independently in controlling parasitaemia, so lower level combinatorial effects cannot be excluded, but it may be prudent to remove very low responders to limit the amount of uninformative data and the risk to a volunteer from whom the expectation might be that the information derived from CHMI would not be useful in designing future vaccines (in as much as this can be predicted).

 We agree with the reviewer that these are important and complex questions. Our approach will be to ensure that the volunteers enrolled have a range of responses to schizont extract as a proxy for other responses, and to then examine the range of measured responses to recombinant antigen and functional assays for both linear trends and thresholds. The functional work proposed will not be used to select individuals for enrolment, but rather to correlate infection outcome with functional and antibody responses. Since adults in our setting have a very low rate of febrile malaria episodes following universal exposure to malaria in childhood, we do not expect uninformative very low responders to be present in our population. We agree that combinatorial effects are important to study.

The following paragraph has been added to the assessment of immunity to malaria section which highlights especially the issue of combinations with Osier et al 2014 as an example of combinations of antigen analysis: “In further exploratory analysis we will also undertake principal component analyses to determine if there are typical “signatures” of protective responses, and analyses for combinations of protective antigens as previously described by Osier et al 2014. The statistical significance of protective antigen combinations will be tested using interaction terms and summary metrics as in previous studies [Osier F et al 2014, Bejon P et al 2011].”

1. An appendix describing the antigens to be used for screening volunteers would be useful. Will different variants of proteins be incorporated or just those for NF54 (the challenge strain)?

We have added the reference to the recently published KILCHIP paper Kamuyu et al 2018 describing the antigens. We anticipate many further analyses internally and with external parties which are yet to be defined.

1. The range of analytical tools to be used to measure various aspects of host immunity is impressive, including phenotypic and transcriptional approaches.  A table to summarize these assays and provide references to their implementation elsewhere would be helpful in determined their likely efficacy.  Many of the assays are relatively common, but others are less so, at least to me (e.g. impact on dendritic cells; sporozoite inhibition).

 We now include a table (Table 7) to summarise the potential assays to be utilised and their respective references. In addition, the sample set generated will be made available to external parties for further study.

 

1. How will the database cope with the different forms of information?

The size of the database will be comparable to previous multidimensional immunological work (ref https://www.ncbi.nlm.nih.gov/pmc/articles/PMC4856877/ and https://www.ncbi.nlm.nih.gov/pmc/articles/PMC3126844/ *)*  and our standard in-house data handling platforms (such as OpenClinica and LabKey) will be sufficient to manage the various datasets.

1. Does the power calculation take into account the multiplicity of parameters? 

 The power calculation considers multiple variables, including a Bonferroni correction for 100 comparisons. We calculate n=200 for 80% power to find a single variable with a correlation coefficient of 0.3.

 

1. Several of the authors have been involved in complex cohort studies on malaria immunity in Kilifi – have these generated an analytical framework that could be applied to this study?

There will be a deliberate analytical framework generated and applied to the data generated. This will be largely guided by the outcomes that will be observed from parasite qPCR results. For instance, for parasite growth rates and liver-to-blood inoculums (i.e. the number of parasites exiting the liver and infected red blood cells), these will be calculated by fitting established models to quantitative PCR data. The growth rate will be regressed against the individual immunological measures such as intensity and/or breadth of antibody responses to parasite antigens. Given the co-linearity of responses, a multivariable model is likely to be more informative in testing our hypotheses that: naturally acquired immunity to malaria depends on responses to specific identifiable antigens; and the overall intensity and/or breadth of antibody responses to parasite antigens are associated with immunity. Rather than simply enter all variables in a single multivariable model, we will consider entering variables in clusters and take forward the independent predictors to a final multivariable model. These clusters will be pre-defined and be based on: (a) biological links (for instance grouping all red cell surface antigens separately from merozoite antigens); and (b) on co-linearity (i.e. on clustering groups of antigens with greatest cross-correlations). We will score breadth as the number of antigen responses in the top quartile and mean intensity as the mean of normalized responses for each antigen. These scores will be determined separately for merozoite antigens and for red-cell-surface antigens. We will also incorporate functional assays, anti-circumsporozoite antibody levels, responses to schizont extract, and scores for mean intensity and breadth of response across antigens which will be added to the final model to test the hypotheses mentioned above. To test the hypothesis that there are specific thresholds above which immunological responses control parasite growth, we will examine this final model for non-linearity using multiple fractional polynomials and Hill functions to determine if there are discrete thresholds predicting efficacy according to previously used methods.

We have added text to the section on statistical and analytical plans.

 

1. Why are parasite transcriptomes being performed?  How will these data contribute to identifying immune signatures of protection?  If this is just to differentiate community acquired infection from the CHMI strain NF54, this can be done in a much less complicated way.

 Parasite transcriptomes mainly for looking at var gene changes in relation to immunity as previously described by Abdi et al 2017 (BMC Infect Dis), and also examining commitment to transmission stages as secondary aims.

 

1. Finally, it is good to see the social science research nested within the project and these outcomes will provide useful information.  In terms of reporting this and other information, the authors say that they will make the data publicly available “as rapidly as possible”.  Would they be willing to put a time limit on this?

This will be done in parallel with the primary publications arising from the work. We have already begun to make the output of this work readily available as rapidly as possible (refer to Njue et al 2018 Wellcome Open).
